# Unraveling Causality: Innovations in Epidemiologic Methods

**DOI:** 10.31662/jmaj.2024-0246

**Published:** 2025-04-21

**Authors:** Etsuji Suzuki

**Affiliations:** 1 Department of Epidemiology, Graduate School of Medicine, Dentistry and Pharmaceutical Sciences, Okayama University, Okayama, Japan

**Keywords:** attributable fraction, causal inference, confounding, counterfactual model, covariate balance, etiologic fraction, mediation, sufficient cause model

## Abstract

For several decades, the counterfactual model and the sufficient cause model have shaped our understanding of causation in biomedical science and, more recently, the link between these two models has enabled us to obtain a deeper understanding of causality. In this article, I provide a brief overview of these fundamental causal models using a simple example. The counterfactual model focuses on one particular cause or intervention and gives an account of the various effects of that cause. By contrast, the sufficient cause model considers sets of actions, events, or states of nature which together inevitably bring about the outcome under consideration. In other words, the counterfactual framework addresses the question “what if?” while the sufficient cause framework addresses the question “why does it happen?” Although these two models are distinct and address different causal questions, they are closely related and used to elucidate the same cause-effect relationships. Importantly, the sufficient cause model makes clear that causation is a multifactorial phenomenon, and it is a “finer” model than the counterfactual model; an individual is of one and only one response type in the counterfactual framework, whereas an individual may be at risk of none, one, or several sufficient causes. Understanding the link between the two causal models can provide greater insight into causality and can facilitate the use of each model in appropriate contexts, highlighting their respective strengths. I will briefly present three topics of interest from our research: the relationship between the concepts of confounding and of covariate balance; distinctions between attributable fractions and etiologic fractions; and the identification of operating mediation and mechanism. It is important to scrutinize observed associations in a complementary manner, using both the counterfactual model and the sufficient cause model, employing both inductive and deductive reasoning. This holistic approach will better help us to unravel causality.

## Seeing the Invisible – How?

Causality is central to clinical and epidemiologic studies. Without an understanding of cause-effect relationships, we cannot answer basic questions such as “Does this treatment harm or help patients?” or “Does exposure to this material have any effects on our health?” These are causal questions, and humans are innately interested in these types of questions. From birth, we continually gain knowledge about cause-effect relationships around us, sometimes through undesirable experiences, and strive to apply this knowledge to improve our lives and health. Simply put, humans are by nature inquisitive about causality.

In the era of data science, there is growing interest in using data to infer causation in biomedical science, and in this context, artificial intelligence is also of increasing interest. Rapid progress has been made in concepts and methods for causal inference, leading to innovations in epidemiologic methods that address causal questions in biomedical science. However, as expressed by the axiom “association is not causation,” this is not always an easy task. Furthermore, in most situations, causes in biomedical science are neither necessary nor sufficient.

To grasp the nature of causal inference from data, it is important to understand the underlying, though unobservable, structures behind the observed data. How can we see the invisible? Understanding formal causal models is key ^(1)^. For several decades, the counterfactual model and the sufficient cause model have shaped our understanding of causation in biomedical science ^(2), (3), (4), (5), (6)^, and more recently, the link between these two models has enabled us to obtain a deeper understanding of causality ^(7), (8), (9), (10), (11), (12)^. In this article, I provide a brief overview of these fundamental causal models using a simple example, highlighting how innovations in epidemiologic methods contribute to unraveling causality.

## A Hypothetical Example

**Figure 1.**
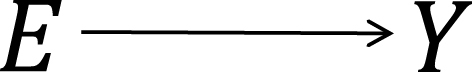
A causal diagram of an exposure *E* and an outcome *Y*.

As an illustration, I use a hypothetical cohort study in Table 1. I let *E* denote a binary exposure of interest (1 = exposed, 0 = unexposed) and *Y* a binary outcome (1 = outcome occurred, 0 = outcome did not occur). The prevalence of exposure *E* is 0.6 (i.e., Pr(*E* = 1) = 0.6). We are interested in the causation between *E* and *Y*, which is described as an arrow from *E* to *Y* in Figure 1. (For the meaning of arrows in causal directed acyclic graphs ^(13), (14), (15)^, see Section 2-2 of Suzuki et al. ^(16)^) From the observed information in Table 1, we can readily calculate the risk difference (RD) estimate as Pr(*Y* = 1|*E* = 1) − Pr(*Y* = 1|*E* = 0) = 0.390 − 0.300 = 0.090. Similarly, we can calculate the risk ratio (RR) estimate as Pr(*Y* = 1|*E* = 1)/Pr(*Y* = 1|*E* = 0) = 0.390/0.300 = 1.300. To sum up, there is a positive association between *E* and *Y* in the population, which may imply the presence of causation between *E* and *Y*.

**Table 1.**
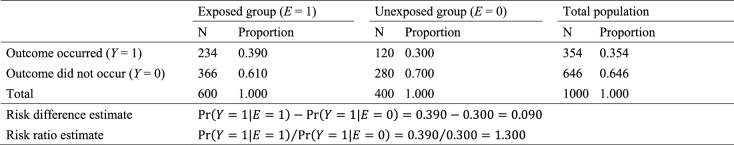
Observed Data in a Hypothetical Cohort Study.

In the following sections, I discuss the underlying data distributions in this cohort study, which, though unobservable in the real world, are important to understand causality. I assume that there is no loss to follow-up and that all variables are measured without errors ^(17)^. For simplicity, I also ignore random errors in this article.

## Through the Lens of Counterfactuals

The counterfactual (or potential outcome) model focuses on one particular cause or intervention and gives an account of the various effects of that cause. This framework addresses the question, “What would have occurred if a particular factor were intervened upon and thus set to a different level than it in fact was?” ^(9)^ Indeed, such “what if?” questions are prevalent in our lives. The idea of conceptualizing causation in terms of counterfactuals can be traced at least as far back as the 18th-century philosopher, David Hume. In his book *An Enquiry Concerning Human Understanding* ^(18)^, Hume has put forward a counterfactual formulation for causation in the following famous passage: “[W]e may define a cause to be *an object, followed by another, and where all the objects similar to the first are followed by objects similar to the second*. Or in other words *where, if the first object had not been, the second never had existed*.” Since then, counterfactual theories of causation have been further developed, and the counterfactual model has become the dominant framework for contemporary causal thinking in epidemiology ^(1), (19), (20)^.

In the counterfactual framework, I let *Y^e^* denote the outcome that would have occurred if, possibly contrary to fact, there had been interventions to set *E* to *e*. Because *E* is binary, there are two potential outcomes, *Y*^1^ and *Y*^0^, corresponding to what would have happened to an individual when the person was exposed and unexposed, respectively. Note that *Y*^1^ and *Y*^0^ are also random variables. Throughout this article, I assume that the consistency assumption is met ^(21), (22)^, which implies that the observed outcome for an individual is the potential outcome, as a function of intervention, when the intervention is set to the observed exposure (i.e., *Y^e^* = *Y* if *E* = *e* (*e* = 0, 1)). Therefore, for each individual, we only get to observe the outcome corresponding to the actual exposure; one of the potential outcomes is observed. Meanwhile, another potential outcome remains unobserved. This is called “the Fundamental Problem of Causal Inference” ^(23)^ because the causal effect of *E* on *Y* for a given individual is defined to be present if *Y*^1^ ≠ *Y*^0^.

What is the underlying data structure of the hypothetical cohort study through the lens of counterfactuals? Based on the joint potential outcomes (*Y*^1^, *Y*^0^), individuals can be classified into the following four (= 2^2^) response types ^(4)^:

Type 1 or “doomed” persons: Exposure is irrelevant because outcome occurs with or without exposure (i.e., (*Y*^1^, *Y*^0^) = (1, 1))

Type 2 or “causal” persons: Outcome occurs if and only if they are exposed (i.e., (*Y*^1^, *Y*^0^) = (1, 0))

Type 3 or “preventive” persons: Outcome occurs if and only if they are unexposed (i.e., (*Y*^1^, *Y*^0^) = (0, 1))

Type 4 or “immune” persons: Exposure is irrelevant because outcome does not occur with or without exposure (i.e., (*Y*^1^, *Y*^0^) = (0, 0))

In Table 2, I show underlying hypothetical data in terms of response types. I let *p_j_*, *q_j_*, and *r_j_*, *j* = 1–4, be the proportions of response type *j* in the exposed group, the unexposed group, and the total population, respectively. As described below, the concept of response type is fundamental to causal inference because the causal effect of exposure on disease frequency in a population is determined by the distribution of response types of individuals in that population, and not necessarily by the population distribution of covariates ^(4)^. This point, however, has been relatively underappreciated because, despite its importance, the response type of each individual is unobservable ^(24)^.

**Table 2.**
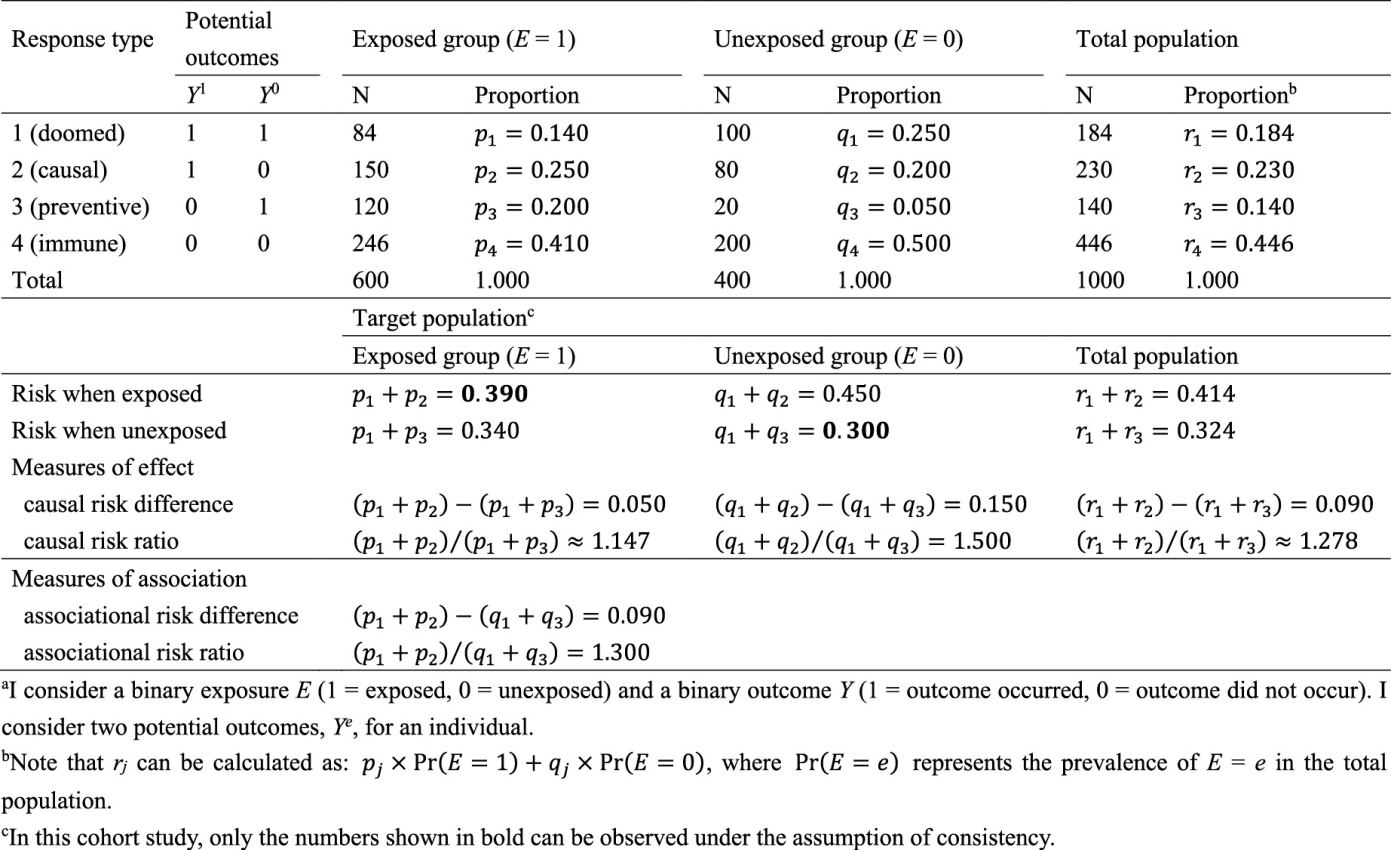
Underlying Hypothetical Data of the Hypothetical Cohort Study in Terms of Response Types^a^.

Recall that, when calculating the RD and RR estimates in Table 1, two conditional probabilities, Pr(*Y* = 1|*E* = 1) and Pr(*Y* = 1|*E* = 0), were used. Among the exposed group, only type 1 and type 2 persons develop the outcome *Y*, and the risk, or incidence proportion, of the outcome in the exposed group is given as Pr(*Y* = 1|*E* = 1) = Pr(*Y*^1^= 1|*E* = 1) = *p*_1_ + *p*_2_ = 0.140 + 0.250 = 0.390. Similarly, among the unexposed group, only type 1 and type 3 persons develop the outcome *Y*, and the corresponding risk is given as Pr(*Y* = 1|*E* = 0) = Pr(*Y*^0^ = 1|*E* = 0) = *q*_1_ + *q*_3_ = 0.250 + 0.050 = 0.300. Therefore, the associational RD is obtained using the proportions of response types as (*p*_1_ + *p*_2_) − (*q*_1_ + *q*_3_) = 0.390 − 0.300 = 0.090, which is equivalent to the RD estimate. Similarly, the associational RR is obtained as (*p*_1_ + *p*_2_)/(*q*_1_ + *q*_3_) = 0.390/0.300 = 1.300, which is equivalent to the RR estimate. These are *measures of association* that can be observed in the real world.

**Figure 2.**
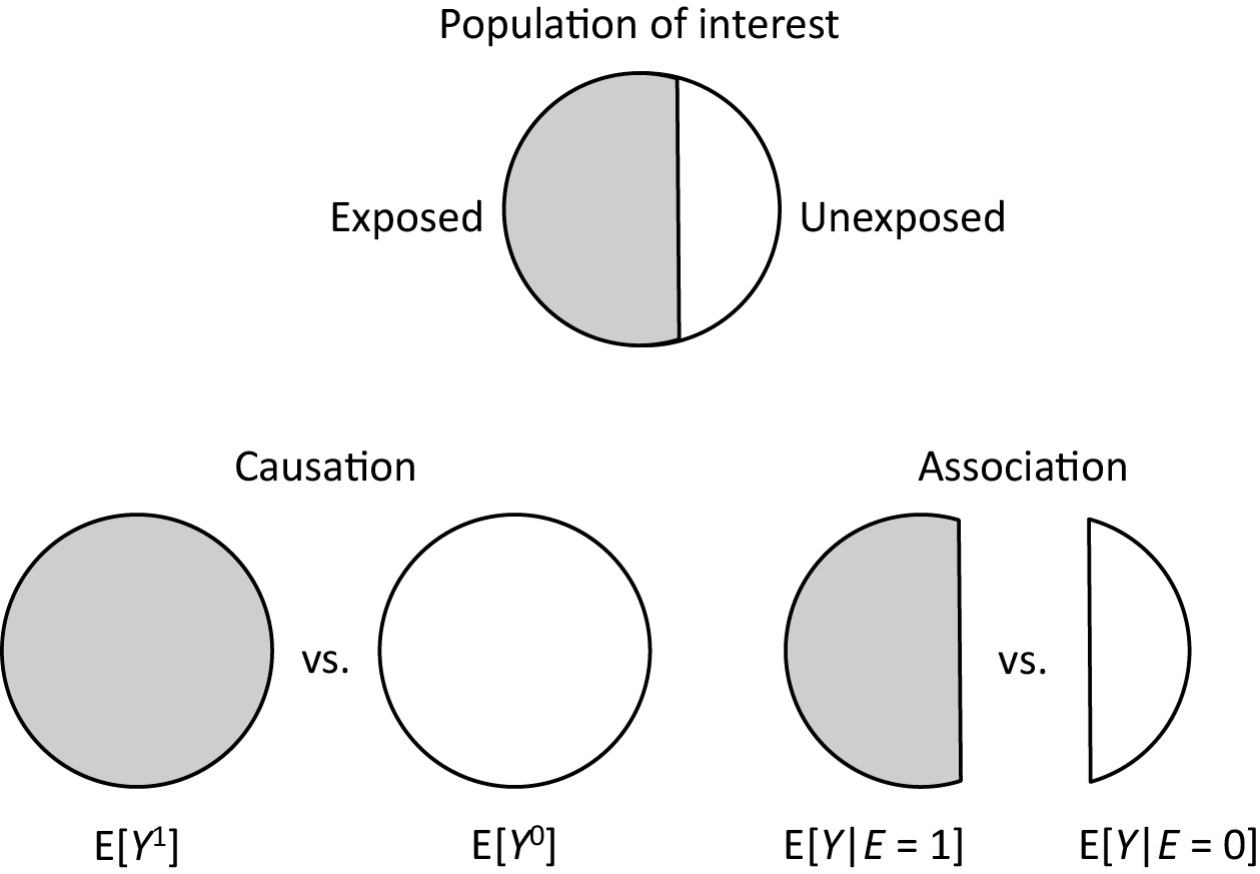
Causation and association in the population of interest. Causation is defined by a contrast of risks in the entire population under two potential exposure values, whereas association is defined by a contrast of risks in subsets of the population determined by the subjects’ actual exposure value. Adapted from Hernán and Robins ^(19)^ with permission from the authors.

By contrast, an average causal effect of *E* on *Y* in the population is defined to be present if E[*Y*^1^] = Pr(*Y*^1^ = 1) ≠ Pr(*Y*^0^ = 1) = E[*Y*^0^] in the population of interest (Figure 2). Note that these are not conditional but marginal probabilities. Importantly, the concept of the target population plays a key role in causal inference ^(25), (26), (27), (28), (29)^. Target parameters for causal inference, or *measures of effect*, cannot be defined unless the target population is clearly defined. For example, when the total population is used as the target population, the counterfactual risk when everyone in the total population is exposed is given as Pr(*Y*^1^ = 1) = *r*_1_ + *r*_2_. In a similar manner, the counterfactual risk when everyone in the total population is unexposed is given as Pr(*Y*^0^ = 1) = *r*_1_ + *r*_3_. Accordingly, the causal RD in the total population is calculated as (*r*_1_ + *r*_2_) − (*r*_1_ + *r*_3_) = 0.414 − 0.324 = 0.090, whereas the causal RR is calculated as (*r*_1_ + *r*_2_)/(*r*_1_ + *r*_3_) = 0.414/0.324≈1.278.

Once information about these target parameters, or *causal estimands*, is available for the target population, the concept of confounding—or more precisely, *confounding in measure*—becomes clearer by comparing the causal estimands with the corresponding measures of association ^(27), (28), (29), (30), (31), (32), (33), (34)^. If we use RD as a measure of interest when the target population is the total population, the associational RD (i.e., 0.090) is identical to the causal RD (i.e., 0.090), and we say there is no confounding (in measure for RD). However, when either the exposed or unexposed group is used as the target population, there is confounding (in measure for RD); the associational RD is higher than the causal RD in the exposed group (i.e., 0.050), while it is lower than the causal RD in the unexposed group (i.e., 0.150). This exemplifies that the concept of the target population is important when explaining confounding because this depends on the population selected as the target of inference.

However, confounding depends not only on the target population but also on the notions of *confounding in distribution* and *confounding in measure* ^(27), (28), (29), (30), (31), (32), (33), (34)^. As summarized in Table 3 ^(19), (35), (36), (37)^, when the total population is used as the target population, no confounding in distribution is stronger than no confounding in measure. Thus, as shown in our hypothetical cohort study, even when confounding in distribution is present, confounding in measure is not always present. Furthermore, while confounding in distribution is scale-independent, confounding in measure is scale-dependent. In this cohort study, there is no confounding in measure for RD, while there is confounding in measure for RR. When either the exposed or unexposed group is used as the target population, distinguishing between the two notions is subtle because the necessary and sufficient conditions for no confounding become equivalent. Moreover, to clarify a further distinction between the notions of *confounding in expectation* and *realized confounding*, it is useful to examine the distribution of exposure status in the target population ^(27)^. To grasp the explicit distinction between these two notions, we need to understand the “mechanism” that generates exposure events, rather than the “product of that mechanism” ^(27), (31), (38)^. Despite its importance, the differing notions of confounding have not been fully appreciated in the literature, leading to confusion in epidemiology regarding causal concepts. See Suzuki et al. ^(27)^ for further discussion.

**Table 3.**
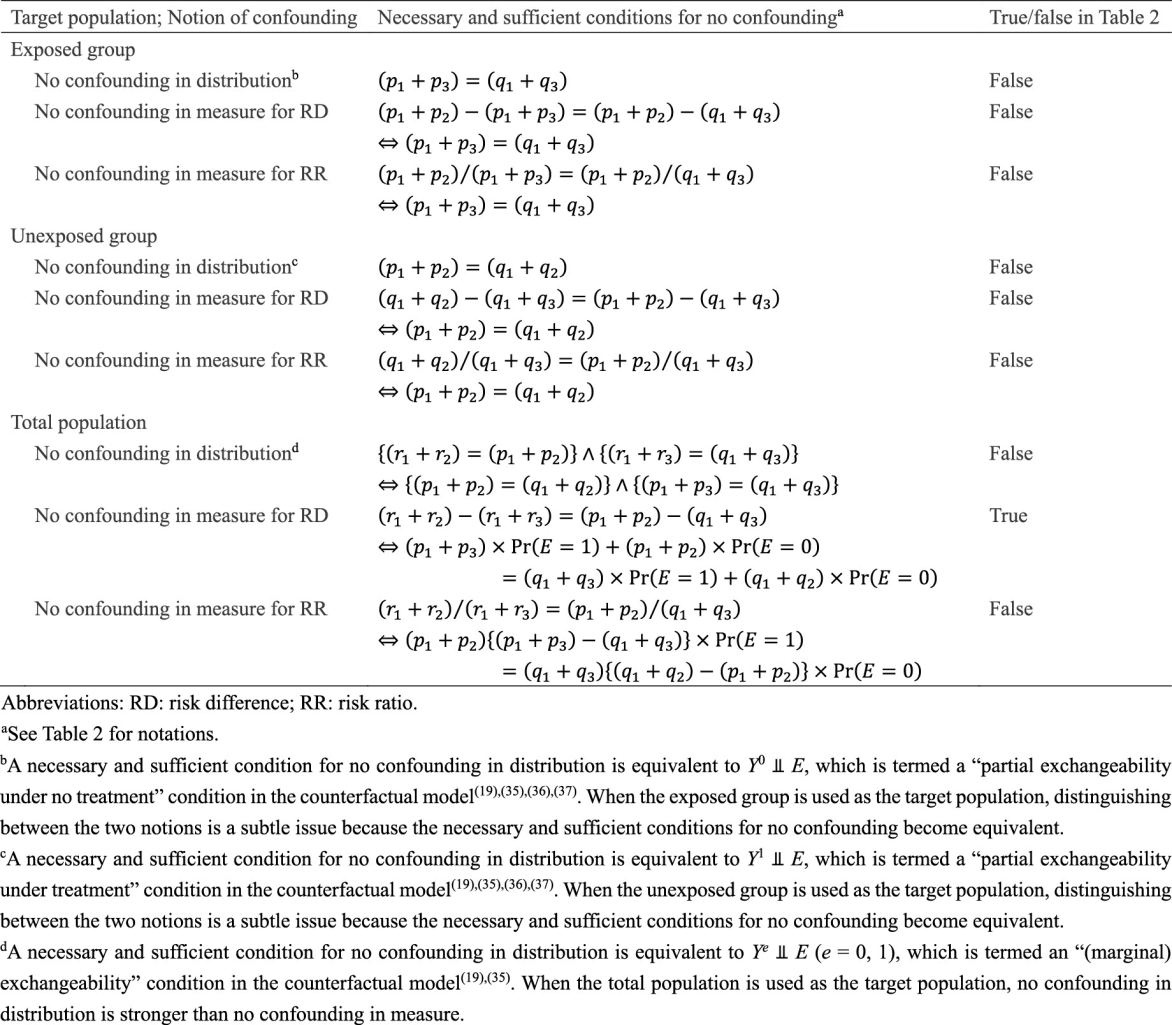
Necessary and Sufficient Conditions for No Confounding.

## Through the Lens of Sufficient Causes

Mechanisms are particularly important in understanding causal processes in biology ^(39)^. Although the meaning of mechanisms may substantially vary across disciplines, careful scrutiny of the concept of the mechanism provides deeper insight into why uncertainty or randomness is so prevalent in biomedical and social sciences ^(40)^. The sufficient cause model was introduced in the field of epidemiology by Rothman ^(3)^, and it has shaped our understanding of causation in biomedical science. Similar models arise in philosophy ^(41)^, law ^(42)^, and psychology ^(43)^.

The sufficient cause model considers sets of actions, events, or states of nature which together bring about the outcome under consideration. In contrast to the counterfactual model, the sufficient cause model gives an account of the causes of a particular effect, addressing the question, “Given a particular effect, what are the various events which might have been its cause?” ^(9)^ Note that the sufficient cause framework and the counterfactual framework address different questions. The counterfactual framework addresses the question “what if?” By contrast, the sufficient cause framework addresses the question “why does it happen?,” which is also a frequently occurring question about causality.

In the sufficient cause model, causation is conceptualized as a collection of different causal mechanisms, each sufficient to produce the outcome. These causal mechanisms are called “sufficient causes,” with each consisting of a minimal set of conditions or “component causes.” Whenever all the component causes of a particular causal mechanism are present, the mechanism operates and the outcome inevitably occurs. Within the philosophical literature, the sufficient cause framework is most closely associated with the work of Mackie ^(41)^, who proposed that when we refer to something as a “cause”, it is then generally known to be an “*insufficient* but *necessary* part of a condition which is itself *unnecessary* but *sufficient* for the result.” From the initial letters of the words italicized above, the term “INUS condition” is used as shorthand for this statement. Following Rothman ^(3)^, I refer to each component of a sufficient cause as a “component cause” or simply a “cause”, in alignment with the concept of an INUS condition. Drawing on Mackie’s terminology ^(41)^, the causal mechanism of interest can be described as “a condition which is itself unnecessary but sufficient for the result.”

**Figure 3.**
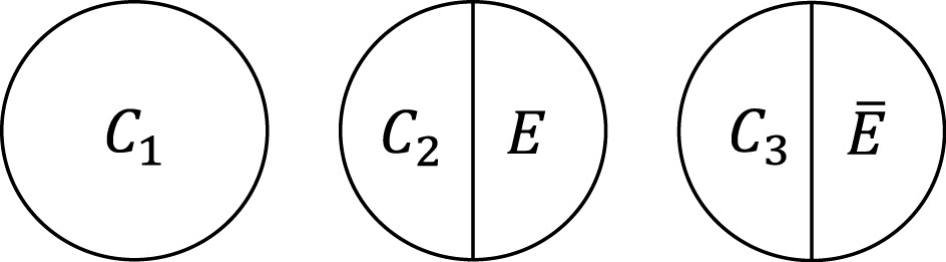
Three types of sufficient causes for *Y*. I consider a binary exposure *E* and a binary outcome *Y*.

Within the sufficient cause framework, we usually include one component cause to represent unspecified events, conditions, and characteristics that must be present or must have occurred at the instance of the outcome of interest. The necessity of the unknown or unmeasured component cause underscores the pervasive uncertainty observed in the biomedical and social sciences ^(40)^. Then, how many types of sufficient causes can we enumerate in the hypothetical cohort study? In the sufficient cause framework, each sufficient cause for the outcome *Y* might require the presence of *E*, the presence of *Ē*, or may not require either, where I let *Ē* denote the complement of *E* in the terminology of events. We could thus enumerate three different types of sufficient causes for *Y* along with certain background factors *C_k_*: *C*_1_, *C*_2_*E*, and *C*_3_ *Ē* (Figure 3). Here, *C_k_* denotes a set of all components or factors, other than the presence of *E* and *Ē*, that may be required for a particular mechanism to operate. Thus, these background factors may comprise several combinations of variables, each of which is part of the sufficient causes. For simplicity, I denote the presence of these background factors as *C_k_* = 1 and their absence as *C_k_* = 0. An individual is at risk of, or susceptible to, sufficient cause *k* if *C_k_* is present for that person.

What is the underlying data structure of the hypothetical cohort study through the lens of sufficient causes? Importantly, an individual may be at risk of none, one, or several sufficient causes. Therefore, based on the patterns of joint background factors (*C*_1_, *C*_2_, *C*_3_), we can enumerate eight (= 2^3^) risk status types as shown in Table 4 ^(44)^. For example, individuals of risk status type 1 are at risk of sufficient causes 1, 2, and 3 (i.e., (*C*_1_, *C*_2_, *C*_3_) = (1, 1, 1)). Table 4 also shows underlying hypothetical data in terms of risk status types. I let *s_j_*, *t_j_*, and *u_j_*, *j* = 1–8, be the proportions of risk status type *j* in the exposed group, the unexposed group, and the total population, respectively. Like the response type, the risk status type of each individual is unobservable.

**Table 4.**
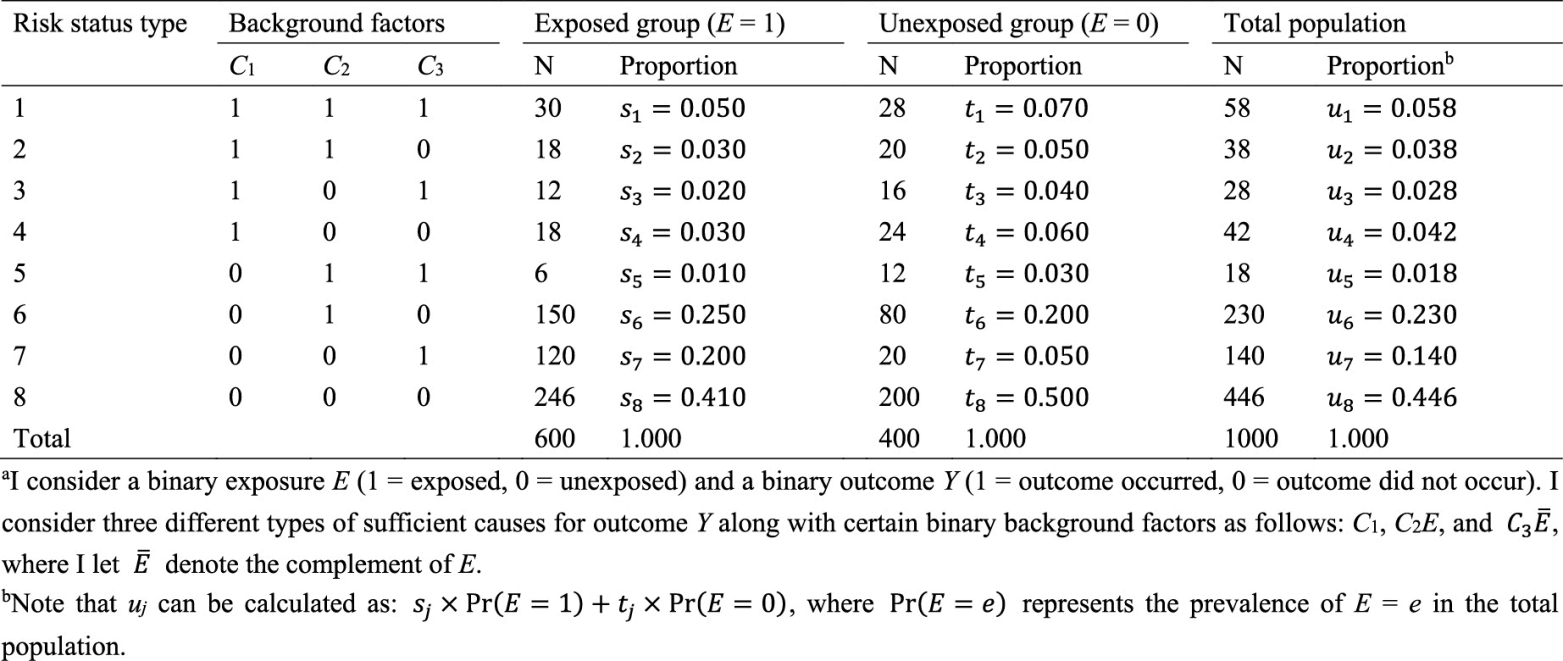
Underlying Hypothetical Data of the Hypothetical Cohort Study in Terms of Risk Status Types^a^.

Importantly, the sufficient cause model makes clear that causation is a multifactorial phenomenon. It is the combination of various conditions that leads to the health or social outcomes that we seek to study. Therefore, in most population health settings, it is not meaningful to try to identify “the” cause; rather we examine “causes” ^(45)^. Furthermore, when many causes or conditions are required for a particular causal mechanism to be operative, elimination of any of them suffices, by definition, to render that sufficient cause inoperative. If multiple conditions are required for a sufficient cause, then they effectively interact within the sufficient cause framework; each needs the other to activate a particular mechanism. We can thus sometimes study and identify the multiple causes that, if eliminated, may be sufficient to substantially reduce the occurrence of a disease. This point is closely related to what I will discuss in the next section.

## The Link between the Counterfactual Model and the Sufficient Cause Model

**Figure 4.**
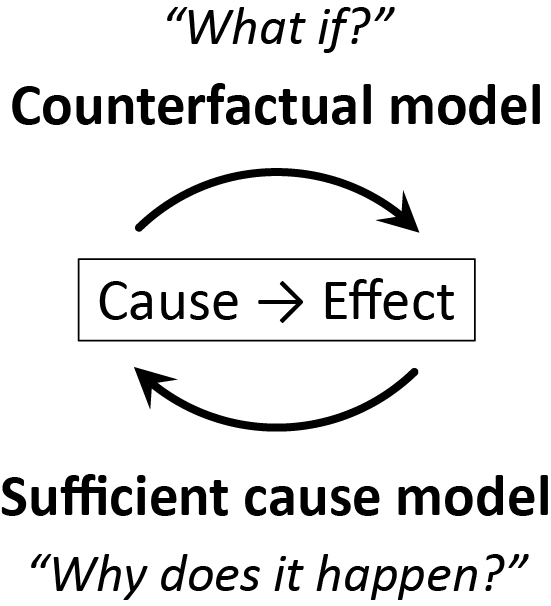
Logical flows of the counterfactual model and the sufficient cause model.

As highlighted above, the counterfactual model gives an account of the various effects or outcomes of a particular cause or intervention, whereas the sufficient cause model gives an account of the causes of a particular effect (Figure 4). Indeed, they are distinct models addressing different causal questions. Importantly, however, these two models are closely related and used to elucidate the same underlying cause-effect relationships ^(46)^, and the link between them has been addressed ^(7), (8), (9), (10), (11), (12)^. In Table 5 ^(11), (44), (47), (48)^, I show a correspondence between the four response types and the eight risk status types, combining the underlying data structure of the hypothetical cohort study through the lens of both counterfactuals and sufficient causes ^(44)^. Linking sufficient causes to the counterfactual model is challenging because of its non-bijective (or more precisely, surjective but not injective) correspondence. Indeed, the sufficient cause model is a “finer” model than the counterfactual model. That is, an individual is of one and only one response type in the counterfactual framework, whereas an individual may be at risk of none, one, or several sufficient causes. This is a fundamental issue when considering the link between these two models. Note that the potential outcomes of *Y* can be described using the background factors *C_k_* as: *Y*^1^ = max{*C*_1_, *C*_2_} and *Y*^0^ = max{*C*_1_, *C*_3_}. For example, max{*C*_1_, *C*_2_} is a binary random variable with a value of 1 unless both *C*_1_ = 0 and *C*_2_ = 0.

**Table 5.**
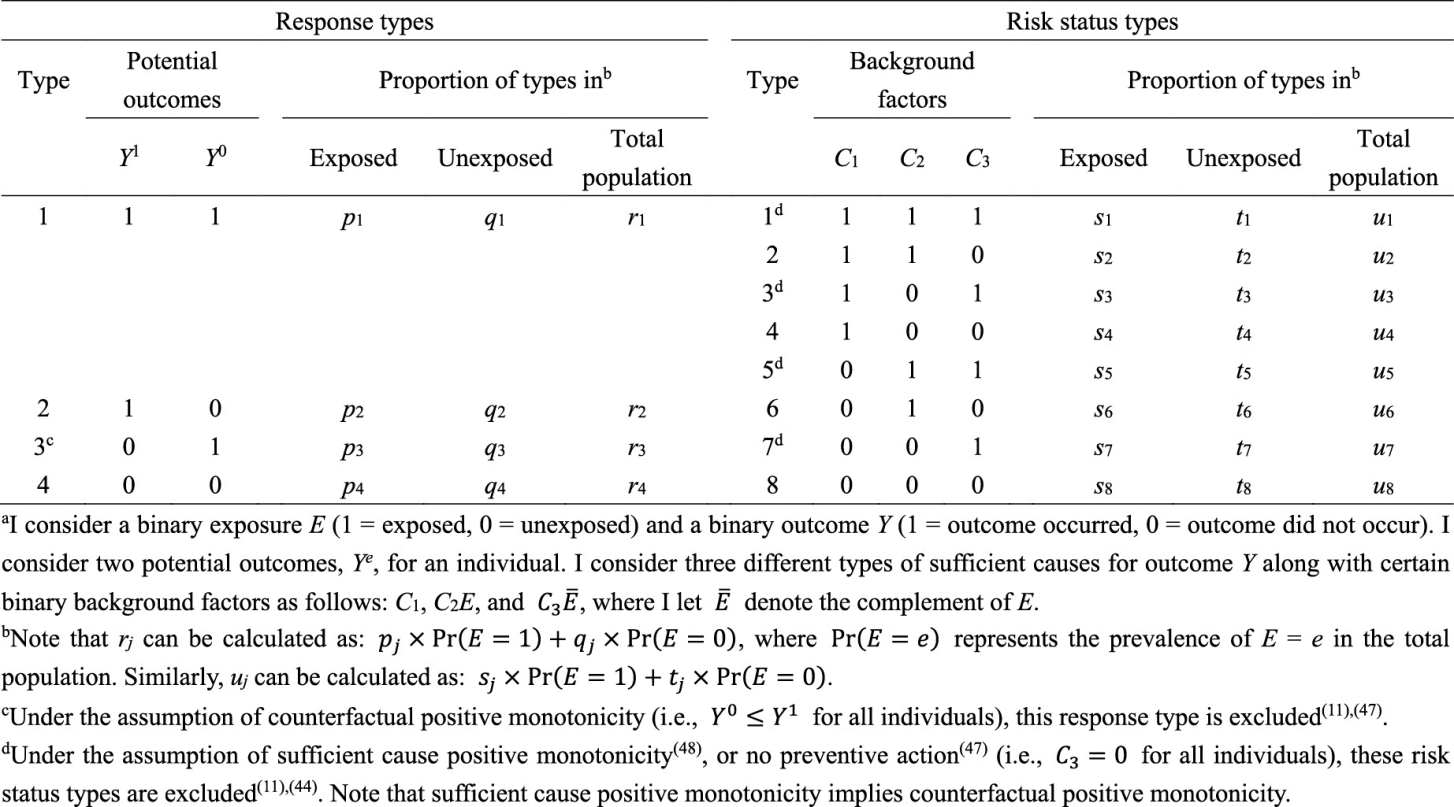
Correspondence between Response Types and Risk Status Types under a Binary Exposure and a Binary Outcome^a^.

Understanding the link between the two causal models can provide greater insight and facilitate the use of each model in appropriate contexts, highlighting their respective strengths ^(49)^. Here, I briefly present three topics of interest from our research. First, the notion of covariate balance was formalized and related to the concept of confounding ^(35), (36)^. We proposed to use each of the background factors in sufficient causes to represent a set of covariates of interest and to examine the presence of covariate balance by comparing joint distributions of the relevant background factors between the exposed and the unexposed groups ^(35)^. Note that a set of covariates can be generally divided into covariates that have no causal coaction with an exposure (i.e., *C*_1_) and those that have causal coaction with an exposure (i.e., *C*_2_ and *C*_3_). Given that sufficient cause 2 (i.e., *C*_2_*E*) contains exposure as a component, it can never complete when the individual is unexposed. Therefore, when the exposed group is the target population, we do not have to consider the comparability of *C*_2_ between the exposed and the unexposed groups. In other words, *C*_2_ represents an irrelevant set of covariates for no confounding, and we need to consider the comparability of only *C*_1_ and *C*_3_ between the exposed and the unexposed groups. Conversely, when the unexposed group is the target population, *C*_3_ represents an irrelevant set of covariates for no confounding because sufficient cause 3 (i.e., *C*_3_ *Ē*) can never complete when the individual is exposed. Thus, we need to consider the comparability of joint distributions of *C*_1_ and *C*_2_ between the exposed and the unexposed groups. Consequently, irrespective of the target population, covariate balance is a sufficient, but not a necessary, condition for no confounding (Table 6) ^(19), (35)^. In other words, confounding implies the presence of covariate imbalance, but not vice versa.

**Table 6.**
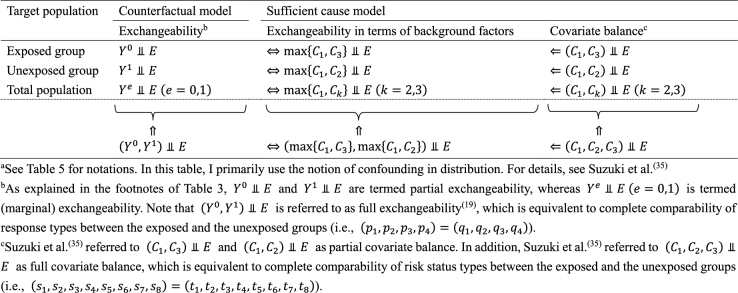
The Relationship between Exchangeability and Covariate Balance for No Confounding^a^.

Second, the link between the two models also provides important distinctions between attributable fractions and etiologic fractions ^(44), (48), (50)^. Since the work published by Doll in 1951 ^(51)^, the attributable fraction has been widely used to assess the potential impact of health interventions in epidemiology. For example, the attributable fraction (population) is generally used when we are interested in the reduction in incidence that would be achieved if the population had been entirely unexposed compared with its current or observed exposure pattern ^(48), (52), (53), (54)^. This is defined in the counterfactual framework as ^(19), (44), (55)^:


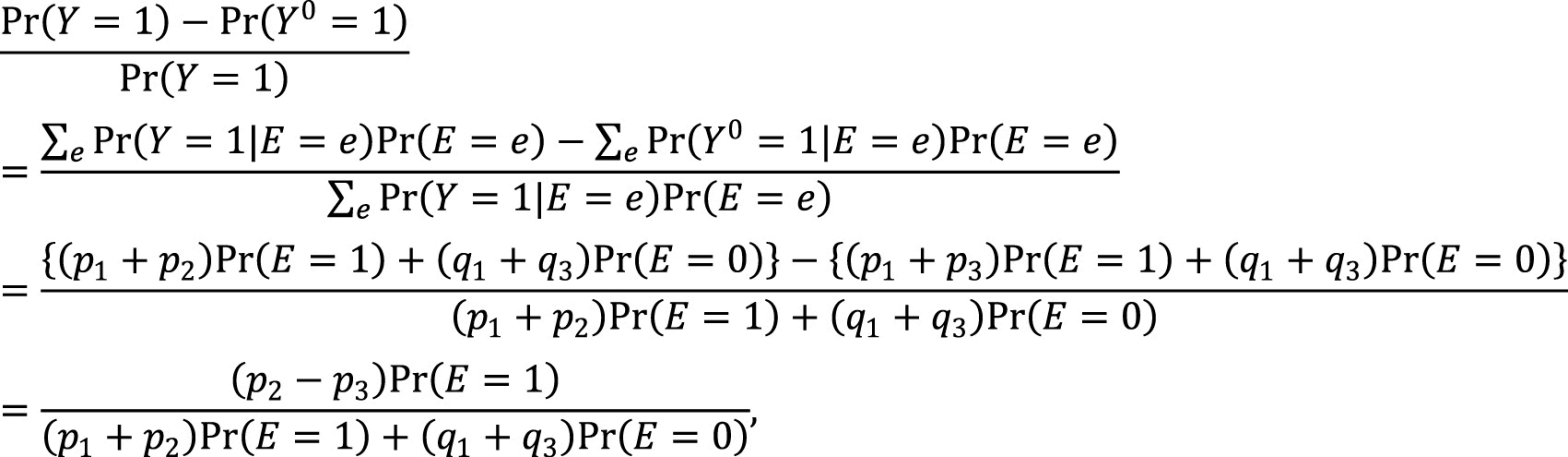


which is approximately calculated as 0.085 in the hypothetical cohort study. Note that this corresponds to the attributable caseload (population) proposed by Suzuki et al. ^(44)^, who distinguished between the attributable caseload and attributable proportion, both of which compose the broader concept of attributable fraction. The attributable proportion (population) is defined as {Pr(*Y* = 1) − Pr(*Y*^0^ = 1, *Y* = 1)}/Pr(*Y* = 1), which ranges from 0 to 1 ^(44)^, and is approximately calculated as 0.424 in the hypothetical cohort study. By contrast, the etiologic fraction has been broadly defined as the fraction of cases that were “caused” by exposure ^(50), (52), (53), (56), (57), (58), (59), (60)^. By taking into account the potential completion time of each sufficient cause, we showed that, when considering a binary exposure and a binary outcome, individuals can be classified into 24 (= 4!) sequence types as shown in Table 7 ^(11), (44), (47), (48)^, which helps to clarify the two types of etiologic fraction: the accelerating etiologic proportion and the total etiologic proportion ^(44)^. The former refers to the proportion of the diseased for whom the exposure “sped up” the time at which the outcome occurred, whereas the latter refers to the proportion of the diseased for whom the exposure is the “actual cause of the outcome.” The accelerating etiologic proportion (population) and the total etiologic proportion (population) are defined as ^(44)^:


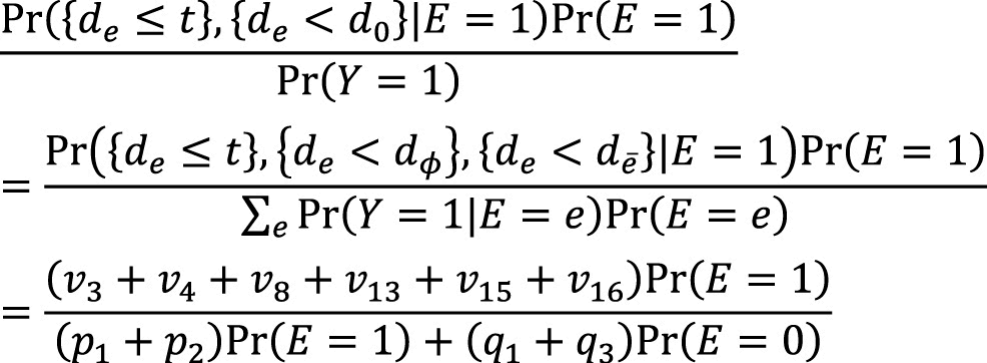


and


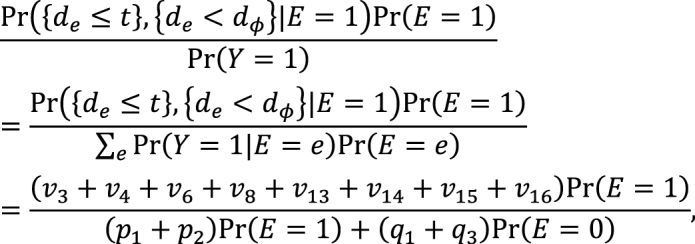


respectively. The latter includes two more sequence types (types 6 and 14) in the numerator, in which *d_e_* is longer than *d_ē_*, and thus equal to or larger than the former. These measures cannot generally be inferred from epidemiologic data, and a lower bound can be calculated by the attributable caseload (population) if it yields a positive value ^(44)^. Although the differences between the two types of etiologic fraction might be subtle, they are closely related to the definition of causality. Therefore, it is important to clarify which measures are used on each occasion. Our recent articles provide further discussion of the attributable fraction and related measures ^(55), (61), (62), (63)^.

**Table 7.**
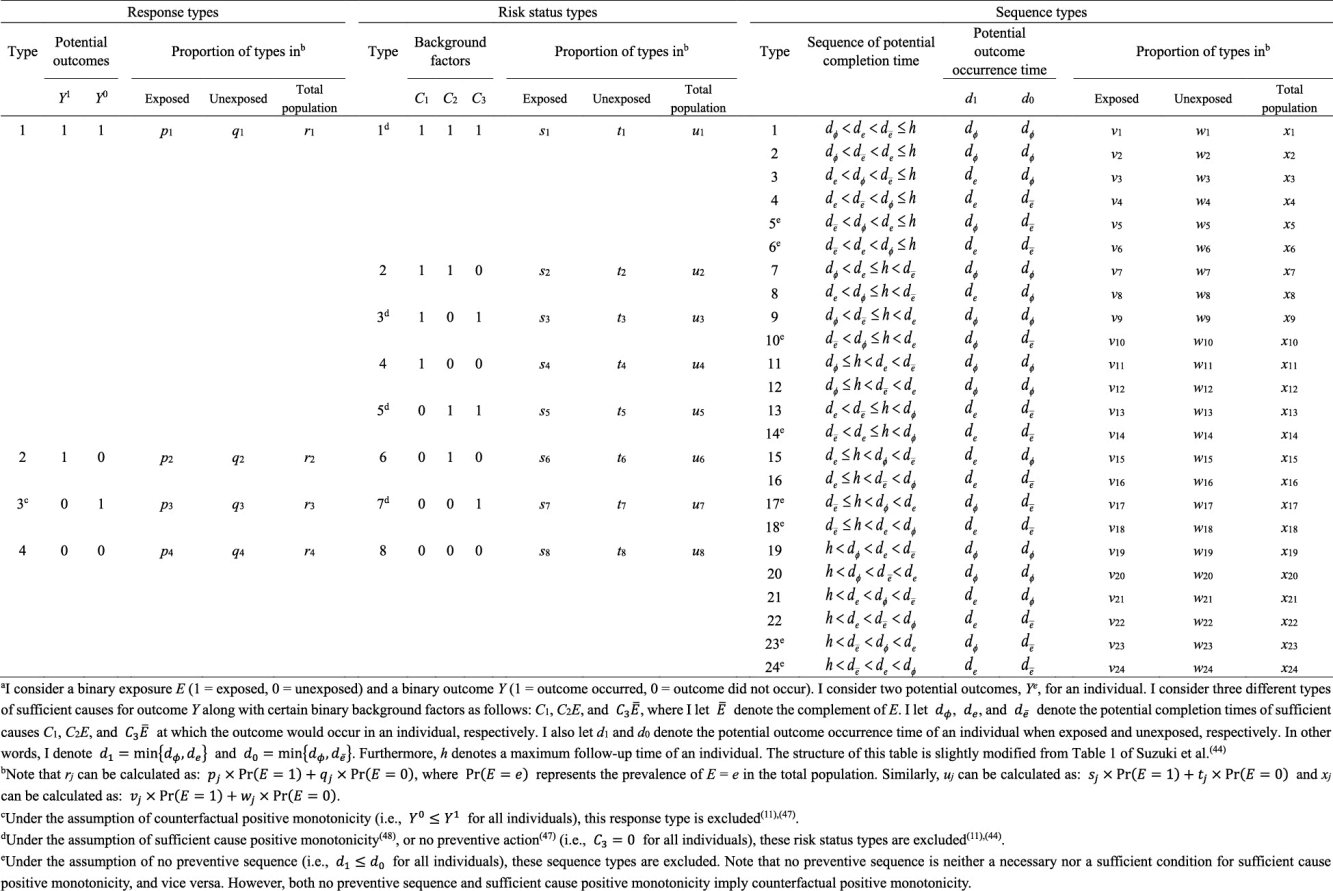
Correspondence between Response Types, Risk Status Types, and Sequence Types under a Binary Exposure and a Binary Outcome^a^.

**Figure 5.**
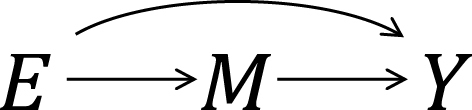
A causal diagram of an exposure *E*, a mediator *M*, and an outcome *Y*.

**Figure 6.**
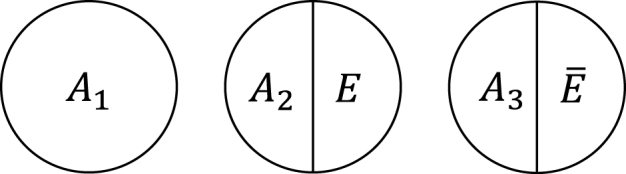
Three types of sufficient causes for *M*. I consider a binary exposure *E* and a binary mediator *M*. Under the assumption of sufficient cause positive monotonicity of *E*, I consider only *A*_1_ and *A*_2_*E*.

**Figure 7.**
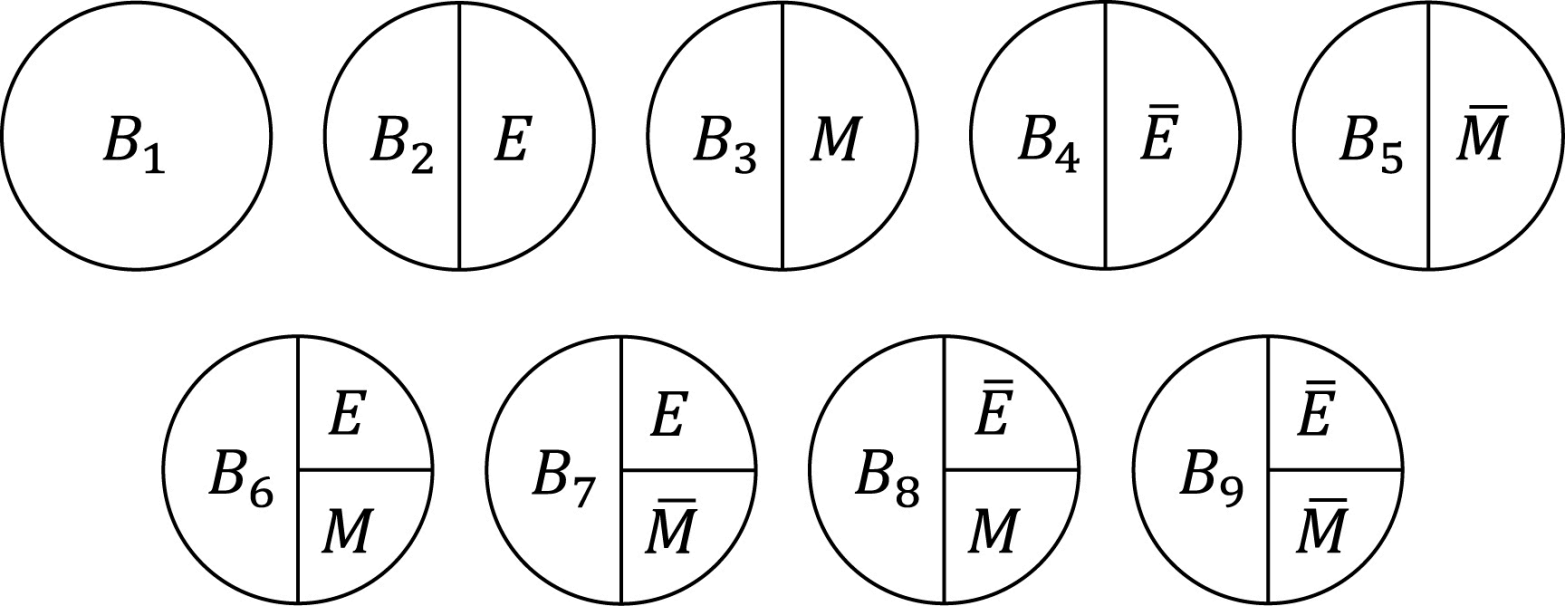
Nine types of sufficient causes for *Y*. I consider a binary exposure *E*, a binary mediator *M*, and a binary outcome *Y*. Under the assumption of sufficient cause positive monotonicity of *E* and *M*, I consider only *B*_1_, *B*_2_*E*, *B*_3_*M*, and *B*_6_*EM*.

**Figure 8.**
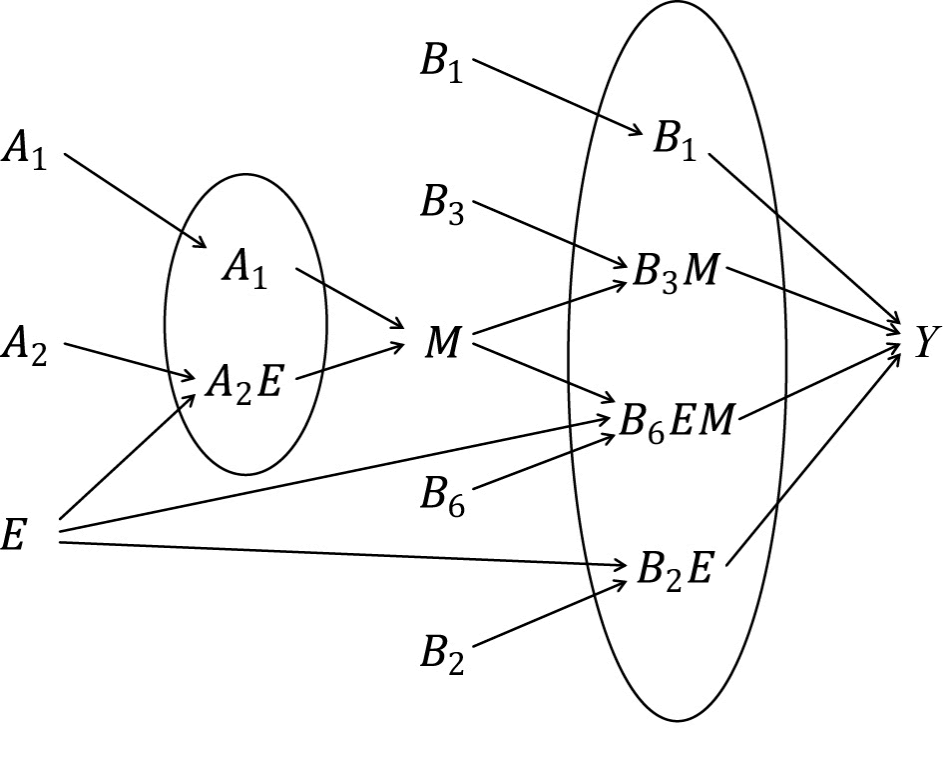
Mediation and mechanism in a causal diagram with a sufficient causation structure. I consider a binary exposure *E*, a binary mediator *M*, and a binary outcome *Y*, under the assumption of sufficient cause positive monotonicity of *E* and *M*. See Figures 6 and 7.

Third, the sufficient cause model has been extended to and used to elucidate the phenomenon of mediation ^(64), (65), (66), (67)^. The assessment of mediation is one way to explore cause-effect relationships in more depth, providing a stronger test for and explanation of the observed associations ^(68), (69), (70), (71), (72)^. In the context of a binary exposure *E*, a binary mediator *M*, and a binary outcome *Y* (Figure 5), I let *M^e^* denote the potential outcomes of *M* if, possibly contrary to fact, there had been interventions to set *E* to *e*. In addition, I let *Y^em^* denote the potential outcomes of *Y* if, possibly contrary to fact, there had been interventions to set *E* to *e* and to set *M* to *m*. By using the compound potential outcomes (or nested counterfactuals), the total effect of *E* on *Y* in the population of interest is given as E[*Y*^1*M*^1^^] − E[*Y*^0*M*^0^^] on the RD scale, or equivalently E[*Y*^1^] − E[*Y*^0^], by making a composition assumption (i.e., *Y^e^* = *Y^eM^e^^*) ^(73), (74)^. As has been well appreciated in the causal mediation literature ^(68), (73), (74), (75)^, the total effect can be decomposed into the pure direct effect (PDE) (i.e., E[*Y*^1*M*^0^^] − E[*Y*^0*M*^0^^]) and the total indirect effect (TIE) (i.e., E[*Y*^1*M*^1^^] − E[*Y*^1*M*^0^^]), or alternatively into the total direct effect (TDE) (i.e., E[*Y*^1*M*^1^^] − E[*Y*^0*M*^1^^]) and the pure indirect effect (PIE) (i.e., E[*Y*^0*M*^1^^] − E[*Y*^0*M*^0^^]). Under the sufficient cause positive monotonicity of *E* and *M* (Figure 6 and 7), we described how we can identify mediation as well as mechanism by showing their correspondence with direct and indirect effects in the counterfactual framework (Table 8) ^(66)^. We defined both mediation and mechanism by explicating their presence and operation in the sufficient cause framework. For example, we define that mediating paths (i.e., *E* → *A*_2_*E* → *M* → *B*_3_*M* → *Y* and *E* → *A*_2_*E* → *M* → *B*_6_*EM* → *Y* in Figure 8) are present in an individual if the individual is at risk of a sufficient cause(s) involving *E* in the *M*-stage and simultaneously is at risk of a sufficient cause(s) involving *M* in the *Y*-stage. Similarly, we define that mediation operates in an individual if the mediating path from *E* to *Y* via *M* is actually activated to make *Y* occur. We demonstrated that, although the PIE implies the presence of mediating pathways, it does not necessarily imply their operation because a non-*M*-mediating path, *E* → *B*_2_*E* → *Y*, may operate to induce *Y*. However, this is not the case for TIE, and non-zero TIE implies the operation, not simply the presence, of mediation. Thus, when assessing mediation, our findings give priority to the TIE over the PIE, exerting influence on practice, even indirectly, by shaping the methods that are available and routinely employed. We also demonstrated how a researcher can decompose the total effect into the effect due to mediated paths and the effect due to non-mediated paths in terms of the probabilities of background factors of sufficient causes ^(66)^. These findings contribute to facilitating looking inside the “black box” between exposure and outcome in epidemiologic studies ^(76)^.

**Table 8.**
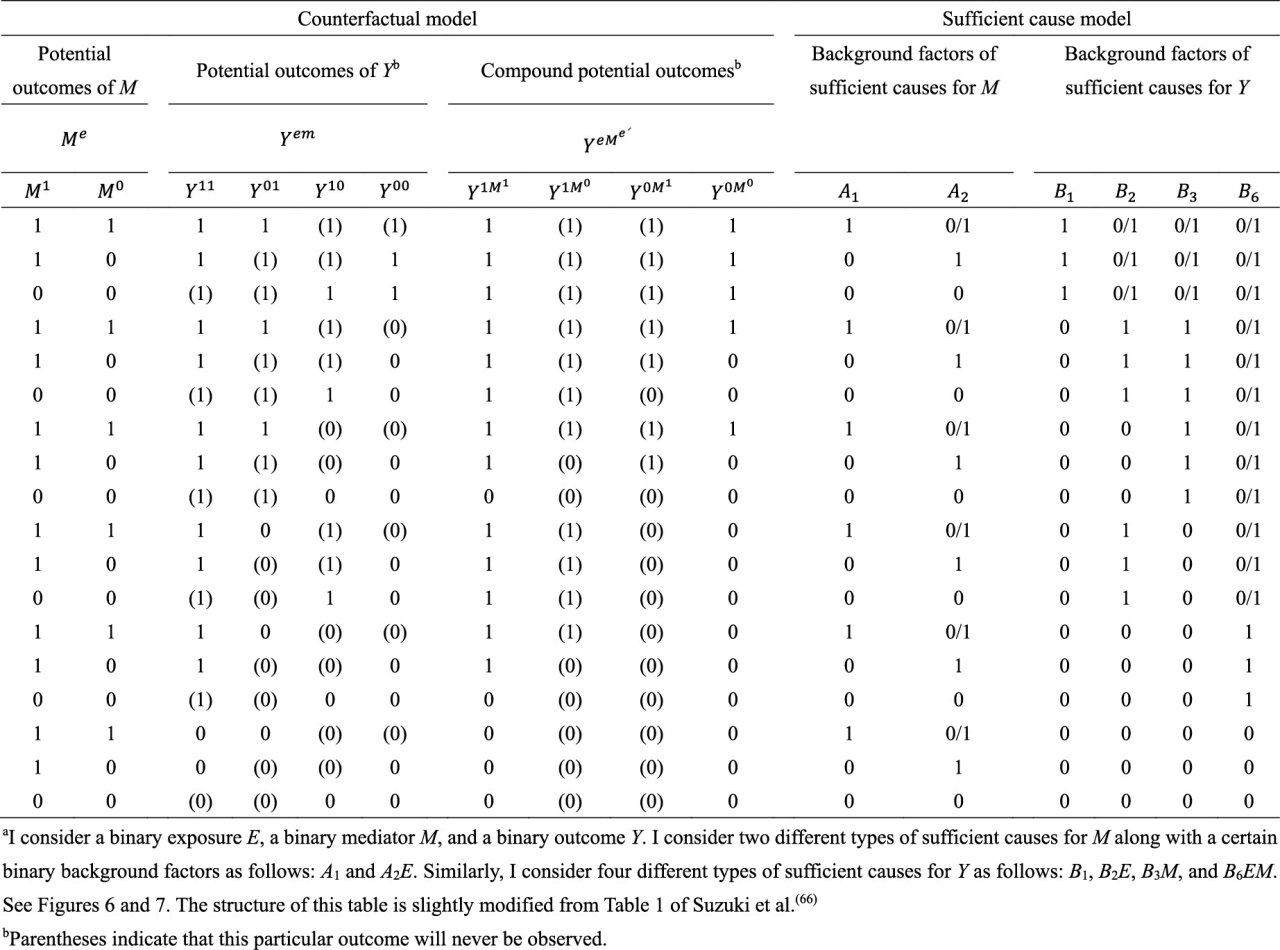
Correspondence between Potential Outcomes and Sufficient Causes under the Assumption of Sufficient Cause Positive Monotonicity in the Context of Mediation^a^.

## Further Remarks

The counterfactual model and the sufficient cause model have become cornerstones for causal thinking in epidemiology ^(2), (3), (4), (5), (6)^. Meanwhile, for decades, attempts have been made in the field of epidemiology to use a set of facts to distinguish causal from noncausal explanations ^(77), (78), (79), (80), (81)^. One well-known approach is the nine viewpoints proposed by Sir Austin Bradford Hill in his President’s Address to the Royal Society of Medicine in 1965 (Table 9) ^(52), (82), (83)^. Unfortunately, the list is often erroneously referred to as the “Bradford Hill criteria” or “causal criteria”, although he warned that there are no “hard-and-fast rules of evidence” for causation ^(83)^. Indeed, despite their popularity, the nine Bradford Hill viewpoints do not provide a set of sufficient criteria to distinguish causal from noncausal explanations ^(84)^, and temporality is a *sine qua non* for causal explanations of observed associations ^(81), (85)^.

**Table 9.**
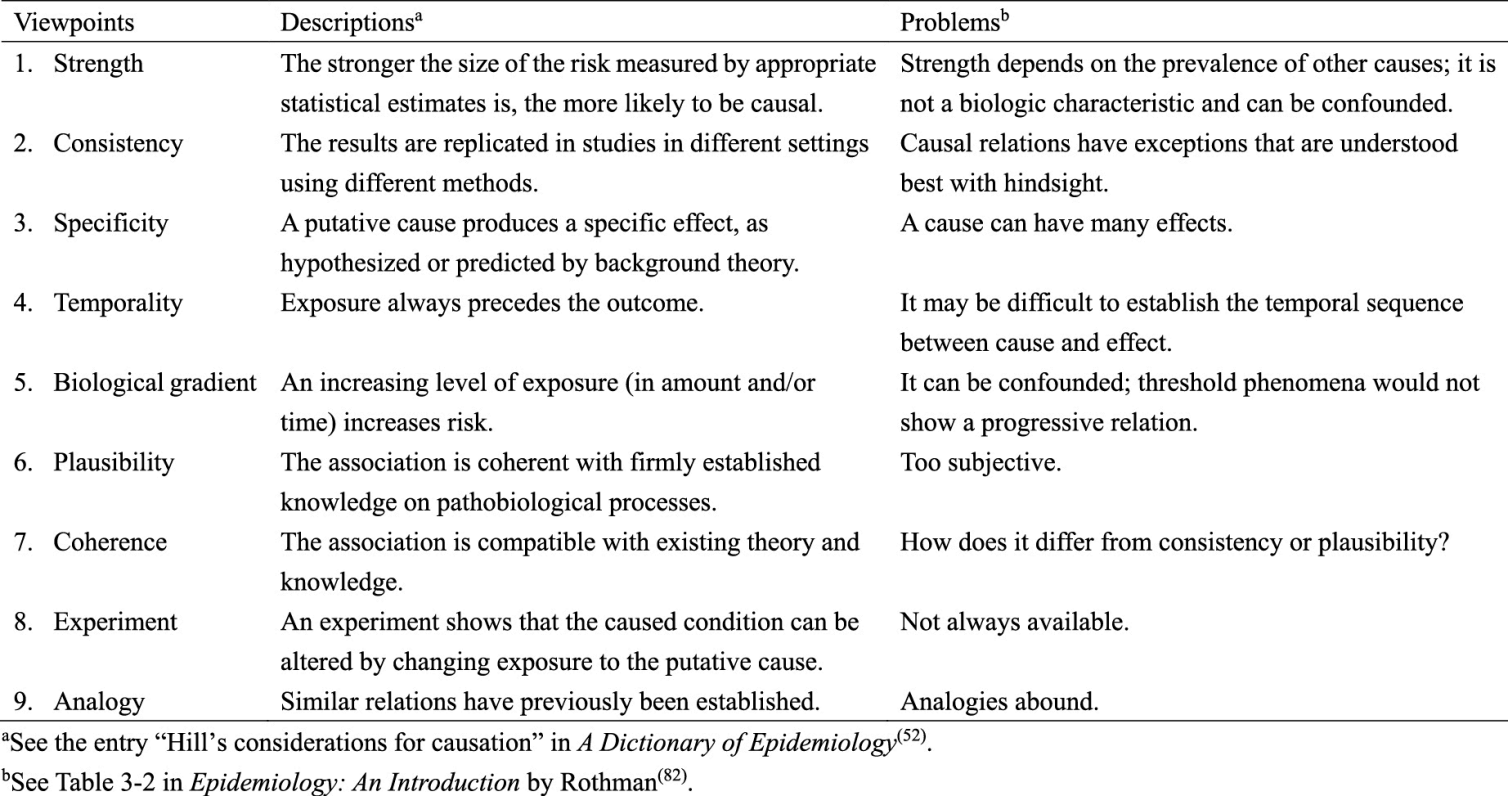
The Nine Bradford Hill Viewpoints.

Although the Bradford Hill viewpoints should not be used as a checklist for evaluating whether an observed association can be interpreted as causal, they are still helpful in assessing causality from an inductive standpoint. However, as emphasized in this article, it is also important to use a deductive form of logic in the formal causal models to draw causal conclusions, by clarifying the premises to be evaluated ^(86)^. Epidemiologic perspectives have been used to shed light on the underlying mechanisms at the genetic, molecular, and cellular levels ^(87), (88), (89), (90), (91)^. To strengthen our assessment in the face of multifactorial causality across multiple levels and dimensions ^(85), (92)^, with time-varying exposures ^(93)^, it is important to carefully scrutinize observed associations in a complementary manner, using both the counterfactual model and the sufficient cause model, employing both inductive and deductive reasoning. This holistic approach will better help us to unravel causality.

## Article Information

This article is based on the study, which received the Medical Research Encouragement Prize of The Japan Medical Association in 2023.

### Conflicts of Interest

None

### Sources of Funding

ES is supported by the Japan Society for the Promotion of Science (JSPS KAKENHI Grant Numbers JP19KK0418 and JP23K09740).

### Acknowledgement

The author thanks Eiji Yamamoto for helpful discussions. The author also thanks Edanz Group (https://jp.edanz.com/ac) for editing a draft of this manuscript.

### Author Contributions

ES conceptualized the study and wrote the manuscript.

### Approval by Institutional Review Board (IRB)

Not applicable.
